# Monitoring the Depth of Sedation During Gastrointestinal Endoscopy: A Narrative Review of Current Evidence and Clinical Recommendations

**DOI:** 10.3390/diagnostics16081245

**Published:** 2026-04-21

**Authors:** Sonia Elena Popovici, Bogdan Miutescu, Stelian Adrian Ritiu, Tudor Voicu Moga, Ioan Sporea, Dorel Sandesc, Ovidiu Bedreag, Marius Păpurică, Mădălina Butaș, Alina Popescu

**Affiliations:** 1 Faculty of Medicine, Victor Babes University of Medicine and Pharmacy, 300041 Timișoara, Romania; popovici.sonia@umft.ro (S.E.P.); miutescu.bogdan@umft.ro (B.M.); sandesc.dorel@umft.ro (D.S.); bedreag.ovidiu@umft.ro (O.B.); papurica.marius@umft.ro (M.P.); alinamircea.popescu@gmail.com (A.P.); 2 Doctoral School, Victor Babes University of Medicine and Pharmacy, 300041 Timișoara, Romania; 3 Anaesthesia and Intensive Care Research Center (CCATITM), Victor Babes University of Medicine and Pharmacy, 300041 Timișoara, Romania; 4 Advanced Regional Research Center in Gastroenterology and Hepatology, Department VII: Internal Medicine II, Discipline of Gastroenterology and Hepatology, Victor Babes University of Medicine and Pharmacy, 300041 Timișoara, Romania; 5 Clinic of Anaesthesia and Intensive Care, Emergency County Hospital “Pius Brînzeu”, 300723 Timișoara, Romania

**Keywords:** gastrointestinal endoscopy, sedation monitoring, depth of sedation, capnography, processed electroencephalogram, bispectral index, patient safety, procedural sedation, endoscopic procedures, multimodal monitoring

## Abstract

Sedation and anesthesia are integral components of modern gastrointestinal endoscopy, enhancing patient comfort and procedural success while adding risks such as respiratory and cardiovascular complications. Accurate monitoring of sedation depth is essential to balance safety and procedural efficacy. This narrative literature review synthesizes current evidence on monitoring depth of anesthesia during endoscopic procedures, including clinical assessment scales, capnography, and processed electroencephalogram (pEEG)-based technologies. The effects of commonly used sedative agents on monitoring parameters and the impact of different monitoring strategies on clinical outcomes are also discussed. Current evidence indicates that clinical assessment remains the cornerstone of monitoring during moderate sedation, while capnography improves early detection of respiratory compromise during deep sedation. pEEG-based monitoring may provide additional value in selected high-risk or prolonged procedures but should complement, not replace, clinical evaluation. A multimodal monitoring approach tailored to sedation depth and patient risk profile is likely to be the most effective strategy for optimizing patient safety. Future research should focus on standardizing monitoring protocols and identifying populations most likely to benefit from advanced monitoring techniques.

## 1. Introduction

Gastrointestinal endoscopy has evolved from an uncomfortable diagnostic procedure to a well-tolerated examination, largely due to advances in sedation and anesthesia techniques [1]. The primary objectives of sedation during endoscopy are to relieve patient anxiety and discomfort, improve procedural outcomes, and diminish memory of the event [1]. However, sedation introduces risks including respiratory depression, cardiovascular instability, and aspiration, necessitating careful monitoring of sedation depth [2].

The concept of sedation depth exists along a continuum, ranging from minimal sedation (anxiolysis) to moderate sedation (conscious sedation), deep sedation, and general anesthesia [3]. Patients may transition between these levels during a single procedure, and individual responses to sedative agents vary considerably. This variability highlights the need for continuous monitoring to maintain the desired level and avoid unintended deeper sedation that could compromise patient safety [4].

Monitoring depth of anesthesia during endoscopy involves both subjective clinical assessment and objective technological measures. Traditional monitoring relies on the evaluation of patient responsiveness, vital signs, and cardiopulmonary parameters. More recently, processed electroencephalogram (pEEG) devices such as the Bispectral Index (BIS) monitor, have been introduced to provide objective, real-time assessment of sedation depth, while capnography allows continuous monitoring of respiratory function [5,6].

This review examines the evidence supporting various monitoring modalities, their clinical applications, and their impact on patient outcomes during endoscopic procedures. It aims to summarize current evidence and provide a practical framework to guide the selection of monitoring strategies based on sedation depth and patient risk.

## 2. Methods

This narrative review was conducted to summarize current evidence regarding monitoring of sedation depth during gastrointestinal endoscopic procedures. Although not designed as a systematic review, the literature search followed a structured approach to ensure comprehensive coverage of relevant studies.

A literature search was performed using PubMed, Scopus, and Web of Science databases for articles published between 2000 and 2025. The following keywords and combinations were used: “endoscopy sedation”, “depth of sedation monitoring”, “bispectral index”, “processed EEG”, “capnography”, and “procedural sedation”.

Studies were included if they met the following criteria:

- Involved adult patients undergoing gastrointestinal endoscopic procedures;
- Evaluated methods for monitoring sedation depth, including clinical assessment tools, capnography, or processed electroencephalogram (pEEG)-based technologies;
- Were clinical trials, observational studies, meta-analyses, or international guidelines.

Studies focusing exclusively on non-endoscopic procedures, pediatric populations (unless highly relevant), or unrelated monitoring techniques were excluded.

The selection of studies was based on relevance to the topic and clinical applicability. Priority was given to high-quality evidence, including randomized controlled trials, meta-analyses, and guideline-based recommendations.

Due to the narrative nature of this review, no formal risk-of-bias assessment or quantitative synthesis was performed.

Although this review follows a narrative approach, priority was given to higher levels of evidence, including randomized controlled trials, meta-analyses, and international guideline recommendations. Observational studies were included when they addressed clinically relevant aspects not adequately covered by higher-level evidence. This approach aimed to provide a structured and clinically relevant synthesis while acknowledging the limitations inherent to narrative reviews.

## 3. Levels of Sedation and Clinical Assessment

The American Society of Anesthesiologists (ASA) and American Society for Gastrointestinal Endoscopy (ASGE) define four levels of sedation based on responsiveness, airway patency, spontaneous ventilation, and cardiovascular function [1]. Because sedation exists on a continuum, it is not always possible to predict how an individual will respond to sedative agents. Practitioners must be prepared to manage deeper-than-intended sedation, including airway and cardiovascular support [7]. The stages are defined by the patient’s responsiveness to stimuli, and transitions between them can occur unpredictably, highlighting the need for continuous monitoring and readiness to intervene [8]. 

Minimal sedation involves normal response to verbal stimulation with unaffected airway reflexes and ventilation. Moderate sedation (conscious sedation) is characterized by purposeful response to verbal or light tactile stimulation, with maintained ventilatory and cardiovascular function. Deep sedation involves purposeful response only to repeated or painful stimulation, with possible need for airway support. General anesthesia results in unarousable patients who often require airway management and may have impaired cardiovascular function [1]. The distinction between these levels is clinically important because different monitoring requirements and personnel qualifications apply to each level. For moderate sedation, the monitoring nurse may perform brief, interruptible tasks, whereas deep sedation requires dedicated, continuous monitoring by personnel not performing the procedure [5].

Among clinical sedation scales, the Richmond Agitation-Sedation Scale (RASS) and the Riker Sedation-Agitation Scale (SAS) are the most reliable and valid for use in critically ill adults, with both demonstrating excellent inter-rater reliability, strong validity, and ease of use across multiple ICU settings [9]. Comparative studies show high correlation and agreement between RASS and SAS, with neither scale being demonstrably superior in ICU practice [10,11,12]. Both scales are suitable for routine sedation monitoring and for determining eligibility for delirium assessment [13,14].

The Ramsay Sedation Scale is widely used and demonstrates moderate reliability and validity; however, it is less granular and less robustly validated than RASS or SAS. Despite these limitations, it remains applicable in both adult and pediatric procedural sedation, including deep sedation for invasive procedures [10]. The Motor Activity Assessment Scale (MAAS) is valid and reliable for mechanically ventilated surgical ICU patients, but its psychometric properties are rated lower than RASS and SAS, and its use is less widespread [10,15,16].

While several sedation scales, including the Richmond Agitation-Sedation Scale (RASS), the Riker Sedation-Agitation Scale (SAS), and the Motor Activity Assessment Scale (MAAS), have been extensively validated in intensive care settings, their routine use in gastrointestinal endoscopy may be limited by the procedural environment. Endoscopic procedures are typically brief, workflow-intensive, and require continuous operator focus, limiting the feasibility of repeated stimulation-based assessments of responsiveness. In this context, monitoring approaches that allow continuous or minimally interruptive assessment may be more practical.

In this context, the Observer’s Assessment of Alertness/Sedation Scale, particularly its modified version, represents a more practical tool for procedural sedation. Unlike ICU-derived scales, the OAA/S allows rapid assessment of patient responsiveness without complex scoring or repeated interruption of the procedure. It has been widely applied and validated in procedural sedation settings, including endoscopy. Consequently, it is commonly used for real-time assessment of sedation depth in endoscopic practice [17].

The Observer’s Assessment of Alertness/Sedation (OAA/S) scale is validated for procedural sedation, particularly with benzodiazepines, and is sensitive to changes in sedation depth. However its psychometric properties are lower than those of RASS and SAS, and it is less commonly used in critical care [17]. In procedural sedation, all tested scales (including modified RASS, Ramsay, and OAA/S) show high reliability and correlation with each other, but their requirement for patient response to stimuli may limit their utility during procedures where movement must be minimized [9,18].

A prospective validation study of 200 patients undergoing endoscopic retrograde cholangiopancreatography (ERCP) compared modified versions of RASS, RSS, and OAA/S scales, using BIS as a reference standard [9]. All three clinical scales demonstrated high reliability with overall Cronbach’s alpha of 0.943 and showed similar correlation with BIS values (Spearman’s correlation 0.673–0.695). However, these scales require patient response to verbal or tactile stimuli, which may interrupt the endoscopic procedure, whereas BIS monitoring provides continuous assessment without patient disturbance [18].

**Figure 1:**
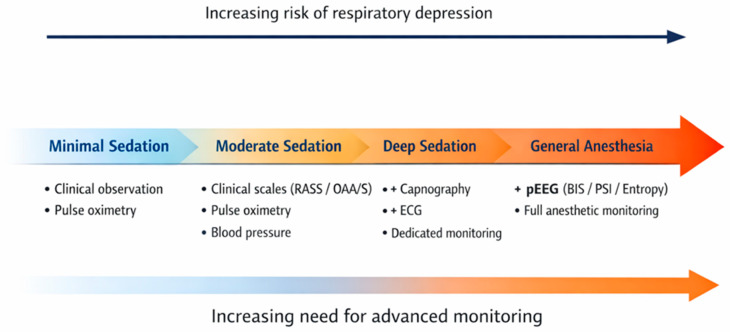
Sedation continuum and corresponding monitoring strategies during gastrointestinal endoscopy.

As the level of sedation progresses from minimal sedation to general anesthesia, the risk of respiratory depression and cardiopulmonary complications increases. Accordingly, the need for advanced monitoring also rises, evolving from basic clinical observation and pulse oximetry to the addition of capnography, electrocardiographic monitoring, and processed electroencephalogram (pEEG)-based technologies (Table 1, Figure 1).

## 4. Standard Monitoring Requirements

### Vital Signs and Cardiopulmonary Monitoring

Current guidelines from the ASGE and ASA establish minimum monitoring requirements for all sedated endoscopic procedures [1,5]. The American Society of Anesthesiologists recommends that during procedural sedation, continuous monitoring of ventilatory function (by observation of clinical signs and, when feasible, capnography), pulse oximetry with appropriate alarms, and regular assessment of blood pressure and heart rate are required. Blood pressure should be measured before sedation and then at regular intervals (typically every 5 min) during the procedure. Electrocardiographic monitoring is integrated in the basic monitoring requirements and should always be used, especially for patients with significant cardiovascular disease or when dysrhythmias are anticipated [7]. The patient’s level of consciousness, ventilatory and oxygenation status, and hemodynamic variables should be recorded before sedation, after administration of sedatives, at regular intervals during the procedure, during initial recovery, and just before discharge. Device alarms must be set to alert the care team to critical changes in patient status. The rationale for these recommendations is that early detection of adverse events (such as hypoxemia or hemodynamic instability) allows for timely intervention, reducing the risk of serious complications during sedation and analgesia [19].

Continuous electrocardiogram (ECG) monitoring is recommended for patients with significant cardiovascular disease or dysrhythmia during moderate sedation [1]. Additional patient populations who may benefit from ECG monitoring include those with significant pulmonary disease, elderly patients, and those undergoing prolonged procedures [5].

Pulse oximetry effectively detects oxygen desaturation and is universally recommended for all endoscopic procedures performed under sedation. Risk factors for hypoxemia include baseline oxygen saturation less than 95%, emergent procedures, prolonged procedure duration, difficult esophageal intubation, and presence of comorbid illness [19]. Supplemental oxygen should be considered for moderate sedation and is required for deep sedation [20]. All guidelines stress the importance of having immediate access to resuscitation equipment and reversal agents, and recommend that any sedation-related adverse event, including airway management or use of reversal agents, be documented [5,20].

## 5. Multimodal Monitoring

### 5.1. Capnography

Capnography is increasingly recognized as a sensitive tool for detecting early respiratory compromise, particularly in deep sedation. The American Society for Gastrointestinal Endoscopy recommends that capnography may be considered for deep sedation, especially in high-risk patients or procedures, as it can reduce the incidence of hypoxemia and apnea [1,5].

Capnography provides noninvasive, real-time assessment of ventilatory function by measuring end-tidal carbon dioxide throughout the respiratory cycle. This technology can detect respiratory depression before oxygen desaturation becomes apparent on pulse oximetry. Sidestream capnography, which samples gas through a nasal cannula, is most commonly used during endoscopic sedation [6].

The role of capnography in endoscopic sedation remains somewhat controversial. For moderate sedation during routine procedures, current evidence does not support mandatory capnography use [5]. A systematic review found inadequate data to support routine capnography during moderate sedation in adults [21]. However, for deep sedation, particularly during advanced procedures such as ERCP, endoscopic ultrasound (EUS), and colonoscopy, capnography may reduce the incidence of hypoxemia and apnea [22].

A meta-analysis of nine randomized controlled trials including 3088 patients demonstrated that capnography significantly reduced the incidence of hypoxemia (odds ratio 0.61, 95% CI 0.49–0.77) and severe hypoxemia (odds ratio 0.53, 95% CI 0.35–0.81) during gastrointestinal procedural sedation [21]. However, there were no significant differences in other outcomes including apnea, assisted ventilation, or changes in vital signs. The American College of Gastroenterology and American Gastroenterological Association recommend that capnography monitoring be considered for patients undergoing endoscopy under deep sedation with propofol [20].

### 5.2. Bispectral Index Technology

The Bispectral Index (BIS) monitor is the most extensively studied pEEG device for monitoring sedation depth during endoscopy. BIS technology analyzes electroencephalographic waveforms from a forehead sensor using a proprietary algorithm to generate a dimensionless number ranging from 0 to 100 [22]. A BIS value between 70 and 90 corresponds to moderate sedation, 60–69 indicates deep sedation, 40–59 represents general anesthesia, and values below 40 denote deep hypnosis [23]. However, in clinical practice, BIS values should not be interpreted as absolute thresholds, particularly in the endoscopic setting. Factors such as patient movement, electrocautery interference, and drug-specific electroencephalographic effects can distort BIS readings, limiting their validity as sole indicators of sedation depth.

Therefore, BIS monitoring should be interpreted as a dynamic trend rather than a single absolute value. Clinical decisions regarding sedation depth and drug titration should be guided by an integrated assessment of patient responsiveness and physiological parameters, rather than relying solely on pEEG-derived indices. This consideration is particularly relevant when using agents such as propofol, dexmedetomidine, or ketamine, which can produce divergent EEG signatures despite comparable levels of clinical sedation.

Several studies have demonstrated correlation between BIS values and validated clinical sedation scales [23,24,25]. Observational studies indicate that a BIS of 80–85 corresponds to optimal sedation during endoscopy performed with opioids and benzodiazepines. However, it is important to recognize that different sedative agents produce distinct EEG patterns at comparable sedation levels, which may affect BIS interpretation. Propofol and dexmedetomidine, for example, produce different EEG spatiotemporal dynamics despite achieving similar clinical sedation depths [24]. Additionally, the performance of pEEG indices may vary depending on the sedative agents used, further limiting their reliability as universal indicators of sedation depth in routine clinical practice.

Multiple meta-analyses have evaluated the clinical utility of BIS monitoring during endoscopic sedation. A 2016 meta-analysis of 11 randomized controlled trials including 1039 patients found that BIS monitoring was associated with reduced total propofol consumption compared to standard monitoring, although the differences in mean propofol consumption were not statistically significant. No significant improvements were observed in recovery time, procedure time, adverse events, or patient and operator satisfaction [23].

A larger 2019 meta-analysis with trial sequential analysis included 13 randomized controlled trials with a total of 1372 patients. BIS monitoring was associated with lower incidences of intraprocedural hypoxia compared to clinical assessment alone, although this finding was not confirmed by trial sequential analysis. Procedure duration, recovery time, and satisfaction scores from both patients and endoscopists were similar between BIS and control groups. The authors concluded that more high-quality, large-scale randomized controlled trials are needed to determine whether BIS monitoring reduces the risk of intraprocedural hypoxia [25].

A prospective study of 200 patients undergoing advanced gastrointestinal endoscopy with target-controlled propofol infusion found that BIS monitoring resulted in significantly lower mean propofol infusion rates compared to monitoring with Modified Observer’s Assessment of Alertness/Sedation scale alone (4.76 ± 1.84 vs. 5.44 ± 2.12 mg/kg/h; *p* = 0.016). Endoscopist satisfaction was higher with BIS monitoring [26].

A prospective study of 1766 patients comparing nurse-administered moderate sedation guided by either Ramsay sedation scale versus BIS monitoring found that BIS-guided sedation was associated with fewer sedation-related adverse events (odds ratio 0.41, 95% CI 0.28–0.62; *p* < 0.0001). Notably, mean BIS values and clinically assessed sedation levels were similar between groups [27]. A 2024 study of 150 patients undergoing fluoroscopic and endoscopic procedures found that BIS guidance allowed real-time assessment of patient response to sedation, predicted safety of further drug administration, and reduced both under- and oversedation, with no patients requiring sedation reversal or airway management [23].

### 5.3. Other pEEG Monitoring Technologies

Other processed electroencephalogram (pEEG) monitoring technologies apart from bispectral index (BIS) include the Patient State Index (PSI, SedLine^®^), Entropy (GE Entropy™), and Auditory Evoked Potentials (AEP, Alaris AEP Monitor). These devices use different proprietary algorithms to quantify the hypnotic component of anesthesia and sedation. They use different algorithms to process raw EEG data but share the common goal of providing objective, continuous assessment of sedation depth [28]. An international expert panel consensus on pEEG monitoring in critically ill patients recommended that frontal pEEG with continuous colored density spectrogram is adequate to monitor sedation level, particularly in patients unfit for clinical evaluation [29].

In a study of 29 older surgical patients, both PSI and BIS demonstrated high sensitivity to anesthetic-induced EEG changes, particularly in detecting periods of EEG suppression (suppression ratio > 25%). Both indices showed significant negative correlations with suppression, indicating reliable detection of deep anesthesia. Entropy also distinguished pre-anesthesia and loss-of-responsiveness states, but PSI and BIS were superior in identifying deep anesthesia with EEG suppression [30].

On the other hand, in a randomized trial of 90 patients, BIS, auditory evoked potentials (AEP), and spectral edge frequency were compared for clinical utility. BIS demonstrated the highest rate of usable signal acquisition, AEP showed the lowest rate of inappropriate readings and the greatest responsiveness to noxious stimuli, and spectral edge frequency had the fastest recovery from signal disturbance. No single monitor was superior in all domains; however AEP demonstrated favorable performance in terms of responsiveness and signal reliability [31].

Entropy monitoring (GE Entropy™) is based on the analysis of EEG signal irregularity and has been validated for distinguishing different depths of anesthesia. It is widely used in Europe and has shown comparable performance to BIS in distinguishing anesthetic states. A large meta-analysis of randomized controlled trials evaluating entropy-guided anesthesia reported a modest reduction in time to awakening compared to standard clinical practice, with a mean difference of approximately 5 min for major surgeries and 3 min for ambulatory procedures. Entropy monitoring is associated with a modest reduction in the use of inhalational anesthetic agents such as sevoflurane; however, evidence for reduced intravenous agent (propofol) consumption and shorter time to readiness for discharge from the post-anesthesia care unit remains of low quality. The incidence of intraoperative awareness was too low to allow meaningful estimation of benefit. Overall, the evidence supporting entropy monitoring for improved recovery and reduced anesthetic use is moderate, and further large studies are needed to clarify its effects on mortality, cost, and clinically relevant outcomes [32].

**Figure 2:**
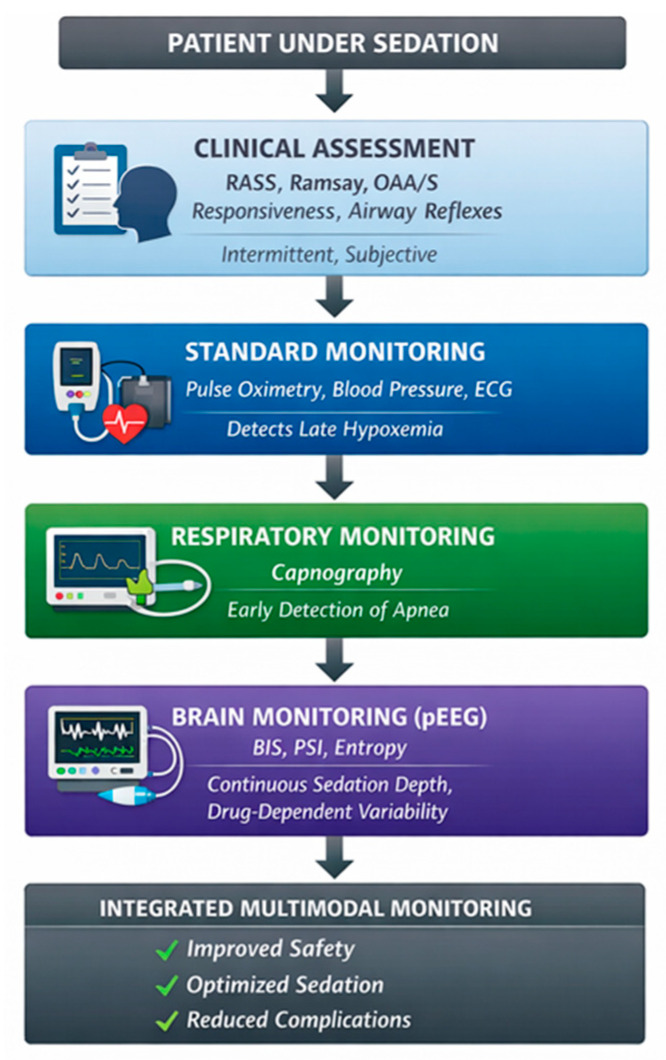
Multimodal monitoring of sedation depth during gastrointestinal endoscopy. Clinical assessment, standard cardiopulmonary monitoring, capnography, and processed electroencephalogram (pEEG) provide complementary information.

The advantages of pEEG monitoring include continuous, objective assessment without requiring patient stimulation, early detection of inadequate or excessive sedation, and potential for improved drug titration. Limitations include cost, need for staff training, variability in performance with different sedative agents, potential for artifact from electrocautery or patient movement, and lack of standardization between different monitoring systems [33]. No large randomized controlled trials have demonstrated clear superiority of one pEEG technology over others in improving major clinical outcomes such as intraoperative awareness or mortality, but all are effective for monitoring depth of sedation [30,31,34] (Figure 2).

While a substantial proportion of the evidence is derived from randomized controlled trials and meta-analyses, some conclusions rely on observational data, which may limit the overall strength of the evidence. Overall, the available evidence suggests that while advanced monitoring technologies provide additional objective data, their impact on major clinical outcomes remains variable, and their optimal use is best achieved as part of a multimodal monitoring strategy rather than as standalone tools (Table 2).

## 6. Sedation Agents and Their Effects on Monitoring

### 6.1. Benzodiazepines and Opioids

The combination of a benzodiazepine (typically midazolam) and an opioid (fentanyl or meperidine) has traditionally been used to achieve moderate sedation during endoscopy. Midazolam is favored for its rapid onset, short duration, lower risk of thrombophlebitis, and strong amnestic effect. Compared to meperidine, fentanyl has more rapid onset and clearance, and is associated with lower incidence of nausea [1].

Benzodiazepines and opioids both depress central nervous system activity and produce dose-dependent changes in clinical sedation scales and processed EEG (pEEG) indices during procedural sedation [35]. Benzodiazepines, such as midazolam, decrease high-frequency EEG activity and increase low-frequency (delta and alpha) power, resulting in lower spectral edge and median frequency values; these changes correlate with deeper clinical sedation scores and are reflected in reduced BIS and entropy values, but the relationship is nonlinear and subject to interindividual variability [36]. Opioids, including fentanyl and remifentanil, primarily provide analgesia but also contribute to sedation. Their EEG effects are less pronounced than those of benzodiazepines and are typically characterized by mild slowing and amplitude reduction. When combined with benzodiazepines, opioids have a synergistic effect, deepening sedation and further lowering pEEG-derived indices [37,38]. pEEG monitors (e.g., BIS and entropy) are sensitive to the sedative effects of benzodiazepines, but their indices may not reliably distinguish between opioid- and benzodiazepine-induced sedation. Consequently, the same pEEG value may correspond to different clinical states depending on the drug combination.

BIS and entropy values exhibit substantial intra- and inter-individual variability when midazolam is co-administered with opioids (e.g., remifentanil, meperidine, morphine, fentanyl), limiting the reliability of pEEG indices for determining sedation depth when used in isolation [39]. The addition of opioids to sedative regimens produces synergistic clinical sedation, whereas BIS and entropy are minimally affected by opioids. This dissociation between clinical sedation scales and pEEG values may result in deeper-than-intended sedation when pEEG targets are used without clinical correlation [40].

The Society of Critical Care Medicine guidelines emphasize that pEEG monitors should not replace clinical assessment, especially when benzodiazepines and opioids are used together, due to high variability and limited predictive value for oversedation or adverse events [41]. Therefore, pEEG monitoring should be interpreted in conjunction with clinical assessment and should not be used as the sole determinant of sedation depth in combination sedo-analgesia protocols. Consistent with this approach, the American Society for Gastrointestinal Endoscopy recommends individualized titration and extended recovery monitoring when benzodiazepines and opioids are used together [38]. This combination is considered safe and effective for upper endoscopy and colonoscopy in patients without risk factors for sedation-related adverse events. The availability of specific antagonists (naloxone for opioids, flumazenil for benzodiazepines) provides a safety advantage, although the effects of reversal agents may be shorter than the effects of the sedatives themselves, necessitating extended recovery monitoring [1].

### 6.2. Propofol-Based Sedation

Propofol has become increasingly popular for endoscopic sedation due to its rapid onset, short duration of action, and favorable recovery profile [42]. Propofol can be administered as monotherapy or in combination with benzodiazepines and opioids (balanced propofol sedation, BPS) [43]. Major studies and meta-analyses consistently demonstrate that propofol is highly effective for procedural sedation, providing rapid onset, reliable procedural conditions, and significantly shorter recovery times compared to traditional sedatives such as benzodiazepines and opioids.

A 2025 Cochrane systematic review of 33 studies with 12,485 participants found that propofol sedation was associated with improved recovery time (mean difference −3.09 min) and higher patient satisfaction compared to traditional sedative agents. There was likely little to no difference in cecal intubation rates and possibly little to no difference in respiratory events requiring intervention [44].

A 2025 network meta-analysis of 152 randomized controlled trials including 26,527 patients found that no regimen demonstrated statistically significant superiority over propofol–opioid combinations for sedation success. However, etomidate–opioid regimens ranked highest for sedation success and overall safety, although they were associated with an increased risk of postoperative nausea and vomiting [45].

A multicenter cohort study in emergency departments found that propofol sedation was associated with deeper sedation, higher procedural success (92% vs. 81%), and shorter sedation duration (10 vs. 17 min) compared with midazolam. Transient apnea was more common with propofol, whereas clinically relevant desaturation occurred more frequently with midazolam. No serious adverse events were reported [46].

Meta-analyses in pediatric populations and gastrointestinal endoscopy confirm propofol’s advantages in recovery time and patient cooperation, with no increase in major cardiopulmonary complications compared to other agents [47]. Systematic reviews in children show rare significant adverse events, with respiratory depression rates up to 1.1% and hypotension requiring intervention up to 5.4% [48].

Propofol lacks intrinsic analgesic properties, which may require higher doses when used alone and increase the risk of deeper-than-intended sedation without a specific reversal agent. Balanced propofol sedation mitigates this limitation by providing analgesia and amnesia with benzodiazepines and opioids, while allowing smaller propofol doses and retaining potential for partial pharmacologic reversibility. Overall, propofol-based sedation is associated with faster recovery, high procedural success, and a safety profile comparable to or better than traditional regimens; however, the risk of respiratory depression and apnea require close monitoring [44,45,46,47,48].

### 6.3. Drug-Specific Considerations for Monitoring

Drug-specific considerations for monitoring during procedural sedation are essential because different sedative and analgesic agents produce distinct effects on both clinical sedation scales and processed electroencephalogram (pEEG) indices. Propofol produces rapid onset and offset of sedation, with characteristic EEG changes including increased frontal alpha and slow-wave activity, and a dose-dependent decrease in BIS and other pEEG indices; however, the relationship between clinical sedation scales and pEEG values is nonlinear, and deeper sedation may not always be reflected by further decreases in pEEG indices once unresponsiveness is reached [49]. Dexmedetomidine induces sedation with EEG patterns distinct from propofol, including increased spindle power and decreased global alpha/beta/gamma power, and may result in lower BIS or PSI values at equivalent clinical sedation levels, complicating direct comparison across agents [50].

A randomized crossover study of 10 healthy volunteers found that, despite similar changes in delta oscillations, dexmedetomidine and propofol produced distinct EEG spatiotemporal patterns at comparable sedation levels. During moderate sedation, dexmedetomidine was associated with decreased global alpha, beta, and gamma power, whereas propofol reduced occipital alpha power and increased global spindle, beta, and gamma activity. These differences in EEG dynamics may contribute to variability in BIS values and reflect distinct underlying sedation mechanisms, highlighting the need to interpret EEG-based monitoring in the context of the sedative agent used [24].

Benzodiazepines (e.g., midazolam) and opioids (e.g., fentanyl, morphine) have additive or synergistic sedative effects; however, opioids alone have minimal impact on pEEG indices. When combined with benzodiazepines, this may lead to clinical oversedation without proportional changes in BIS or entropy values, increasing the risk of unrecognized respiratory depression [41].

Ketamine produces dissociative sedation with electroencephalogram (EEG) features that are distinct from those seen with gamma-aminobutyric acid (GABA) receptor agonist agents [51]. Unlike GABAergic sedatives such as propofol or benzodiazepines, which typically increase low-frequency (delta and alpha) power and suppress higher frequencies, ketamine induces prominent increases in beta (13–30 Hz) and gamma (30–50 Hz) band activity, as well as alternating bursts of slow-delta and gamma oscillations, particularly in the frontal cortex and hippocampus [52]. These oscillatory patterns are associated with the drug’s NMDA receptor antagonism and disinhibition of cortical pyramidal neurons, rather than GABAergic potentiation [53].

During ketamine-induced dissociation and anesthesia, EEG signatures include increased frontal theta and gamma power, decreased alpha power, and alternating periods of high-frequency (gamma) and low-frequency (slow-delta) activity [54]. These features do not correlate linearly with traditional processed EEG indices (such as BIS or entropy), which are calibrated for GABAergic anesthetics and may not reliably track the depth of sedation or dissociation with ketamine [55,56]. Furthermore, the relationship between these EEG changes and clinical sedation scales is inconsistent. Loss of behavioral responsiveness and dissociative states can occur at EEG patterns that would not be interpreted as deep sedation by conventional monitors [57,58].

In summary, the unique EEG features of ketamine—especially increased beta and gamma activity and alternating gamma/slow-delta bursts—do not correlate with traditional processed EEG indices or clinical sedation scales during procedural sedation, limiting the utility of standard EEG-based depth-of-sedation monitors for ketamine-based regimens [51,52,53,54,55,56,57,58].

Recently, newer sedative agents such as Remimazolam have been increasingly used in gastrointestinal endoscopy due to their rapid onset, short duration of action, and favorable recovery profile. As a benzodiazepine, it induces electroencephalographic changes broadly similar to those observed with midazolam, characterized by increased low-frequency activity and attenuation high-frequency components. These changes are associated with decreases in processed EEG indices such as the bispectral index (BIS) and entropy, which may influence the interpretation of sedation depth when relying on pEEG-based monitoring [59].

However, as with other benzodiazepines, the relationship between pEEG-derived indices and clinical sedation depth is variable and may be nonlinear. The use of remimazolam, particularly in combination with opioids, can result in deeper levels of clinical sedation without proportional changes in BIS values, potentially leading to underestimation of sedation depth when relying on pEEG monitoring alone [60]. Therefore, when remimazolam-based sedation is used, pEEG-derived indices should not be interpreted in isolation but integrated with clinical assessment to avoid misestimation of sedation depth. Further studies are needed to define the relationship between pEEG parameters and clinically relevant sedation endpoints under remimazolam, particularly in the context of combination regimens with opioids.

## 7. Adverse Events and Safety Outcomes

### Incidence and Risk Factors

Serious sedation-related complications during gastrointestinal endoscopy are rare but potentially life-threatening [3,4,61]. A large prospective multicenter registry study of 368,206 endoscopies found that major complications occurred in 0.01% of sedated patients, with overall mortality of 0.005%. Minor complications occurred in 0.3% of cases [3].

Independent risk factors for adverse events identified through multivariate analysis include age greater than 75 years, body mass index ≥27, ASA class, emergency procedures, prolonged procedure duration, and use of multiple sedative agents. A retrospective analysis of 23,788 gastrointestinal endoscopies found that age 75 years yielded 46% more adverse events than age 66 years, BMI ≥ 27 resulted in 27% more adverse events than BMI 21, and each one-point increment in ASA score determined a 42% increment in adverse events [61].

The most common adverse events are cardiovascular (hypotension, hypertension, arrhythmias) and respiratory (hypoxemia, respiratory depression, apnea) [2,3,4]. A nationwide retrospective analysis of over 380,000 patients undergoing diagnostic esophagogastroduodenoscopy found acute myocardial infarction occurred at a rate of 1 in 2300 patients and congestive heart failure at 1 in 6700 patients within 30 days of the procedure [4].

Propofol is the widest used anaesthetic drug for procedural sedation. Despite concerns about propofol’s potential for deeper sedation and lack of reversal agent, multiple studies have demonstrated its safety profile is comparable to traditional sedatives. A systematic review and meta-analysis comparing propofol to traditional anesthesia for gastrointestinal endoscopy found similar risk of cardiopulmonary adverse events between propofol and traditional agents. The pooled odds ratio for developing hypoxia with propofol was 0.82 (95% CI 0.63–1.07) and for hypotension was 0.92 (95% CI 0.64–1.32). In non-advanced endoscopic procedures, propofol was associated with 39% fewer complications than traditional sedative agents [62].

An analysis of 428,947 endoscopic procedures from the National Anesthesia Clinical Outcomes Registry found that anesthesia-directed sedation during endoscopy was safe, with serious complications occurring in 0.34% of procedures. Risk factors for serious complications included older age, ASA classes 4 and 5, esophagogastroduodenoscopy (compared to colonoscopy), general anesthesia, overnight shift procedures, and longer case duration [62].

## 8. Special Populations and Clinical Scenarios

Pre-procedural assessment and risk stratification are essential in high-risk patients undergoing endoscopic procedures under sedation and require careful consideration of monitoring strategies. This evaluation should include a detailed history of comorbidities (particularly cardiopulmonary disease, obstructive sleep apnea, and prior sedation-related complications), airway assessment, medication review, substance use, and timing of last oral intake. A focused physical exam should include vital signs, auscultation of the heart and lungs, and airway anatomy assessment. The American Society for Gastrointestinal Endoscopy and the American College of Gastroenterology recommend the use of the ASA physical status classification and, when appropriate, the Mallampati score to stratify risk: higher ASA class and difficult airway are associated with increased risk of sedation-related adverse events. Documentation of the assessment, a procedural pause (“time out”), and adherence to fasting guidelines (minimum 2 h for clear liquids, 6 h for a light meal) are essential to ensure patient safety and quality of care [63].

Identification of high-risk patients should focus on advanced age, significant cardiopulmonary disease, obesity, obstructive sleep apnea, high ASA class (IV/V), anticipated difficult airway, prolonged or complex procedures, and emergency setting. In such cases, involvement of an anesthesia provider is recommended, particularly for those with ASA IV/V status, airway compromise, or when deep sedation or general anesthesia is anticipated [64]. Areas not fully addressed in current guidelines include the routine use of advanced respiratory monitoring (such as processed EEG) and pediatric-specific considerations, which may require additional expertise and resources [61].

Intra-procedural monitoring strategies should be tailored to the patient’s risk profile. At a minimum, all patients require continuous pulse oximetry, non-invasive blood pressure and heart rate monitoring, and visual assessment of ventilation and level of consciousness. In high-risk patients—such as those with significant cardiovascular or pulmonary disease, advanced age, or undergoing prolonged procedures—continuous ECG monitoring is recommended. Supplemental oxygen should be considered during moderate sedation and is required for deep sedation or when hypoxemia is anticipated or develops. Capnography is recommended for deep sedation and for high-risk patients, as it has been shown to reduce the incidence of hypoxemia and apnea in randomized trials. During deep sedation, a dedicated individual should be assigned solely to patient monitoring, and all monitoring data and adverse events must be documented.

Staff training and emergency preparedness are critical. Providers must be specifically trained in the administration of sedation, recognition of sedation-related adverse events, and rescue from deeper-than-intended sedation. Immediate access to resuscitation equipment and medications is mandatory in all settings where sedation is administered [20].

Complex procedures such as ERCP, EUS, and endoscopic submucosal dissection often require longer procedure times and deeper sedation levels. Sedation planning and patient selection must be individualized, taking into account the sedation continuum from minimal sedation to general anesthesia, with doses titrated to achieve a safe, comfortable, and technically successful procedure [42].

Opioid–benzodiazepine combinations are considered safe and effective for routine upper endoscopy and colonoscopy; however, anesthesia provider involvement is recommended for complex procedures or for high-risk patients (ASA IV/V, difficult airway, significant comorbidities, morbid obesity, neurologic disorders, pregnancy, or history of inadequate response to moderate sedation. A review of anesthesia for digestive tract endoscopy noted that advanced invasive procedures require adequate monitoring including capnography adapted to spontaneous ventilation, BIS monitoring, and devices for target-controlled infusion to guarantee quality of sedation and patient safety [65].

## 9. Non-Anesthesiologist Administration of Propofol

The American Society for Gastrointestinal Endoscopy, American Gastroenterological Association, American College of Gastroenterology, and American Association for the Study of Liver Diseases recommend that propofol can be safely administered by non-anesthesiologist providers for procedural sedation in GI endoscopy, provided strict protocols and training requirements are met.

For advanced endoscopic procedures and high-risk patients (e.g., ASA IV/V, difficult airway, significant comorbidities), the societies emphasize careful patient selection, with anesthesia provider involvement recommended for those at increased risk of sedation-related complications. Continuous monitoring of vital signs, including capnography, is required. Endoscopy units must be equipped with resuscitation equipment and reversal agents, and staff must be trained to recognize and manage respiratory depression and other adverse events [42].

Building on these recommendations, recent large-scale studies and systematic reviews have demonstrated that non-anesthesiologist-administered propofol (NAAP) is safe and effective for procedural sedation in appropriately selected patients. In a multicenter retrospective review of over 36,000 endoscopies with nurse-administered propofol, the rate of clinically significant events such as apnea or airway compromise requiring assisted ventilation was less than 0.2%, with no cases of permanent injury or death. Similarly, a prospective study of more than 24,000 patients reported a major adverse event rate of only 0.016%, with minor events (primarily transient hypoxemia) occurring in less than 0.5% of cases. These findings are consistent with randomized controlled trials and meta-analyses, which show that NAAP provides shorter recovery times and higher patient satisfaction compared to traditional opioid–benzodiazepine regimens, without an increase in serious cardiopulmonary complications [66].

For high-risk patients, such as those with ASA class III status, recent data indicate that endoscopist-directed, nurse-administered propofol sedation remains safe, with no increase in major adverse events compared to lower-risk groups. Minor adverse events, such as transient hypoxia or hypotension, are more likely with longer procedures and higher propofol doses, but these events are typically self-limited and manageable with appropriate monitoring and intervention [67]. The American Society for Gastrointestinal Endoscopy emphasizes that NAAP should only be performed by personnel with specialized training in airway management and resuscitation, and that a dedicated individual must be responsible for continuous physiologic monitoring throughout the procedure [1].

It is important to note that the regulatory and legal frameworks governing the non-anesthesiologist administration of propofol vary significantly across countries and healthcare systems. While international guidelines support the safe use of NAAP in appropriately selected patients, implementation is dependent on local regulations, institutional protocols, and credentialing requirements. Therefore, clinicians should ensure compliance with regional policies and training standards when applying NAAP in clinical practice. In some regions, medico-legal constraints continue to limit the use of NAAP in routine practice.

## 10. Future Directions

Despite advances in monitoring technology, important gaps remain in optimizing sedation safety during gastrointestinal endoscopy, particularly regarding the integration of pEEG indices across different sedative regimens. Future research should focus on developing standardized, evidence-based monitoring strategies tailored to sedation depth, procedural complexity, and patient risk profiles.

Emerging technologies such as artificial intelligence (AI)-driven monitoring systems and predictive analytics may enable real-time detection of early respiratory and hemodynamic compromise. Integration of machine learning algorithms with multimodal data sources—such as capnography, pEEG, and vital signs—could enable individualized risk prediction and support timely intervention before clinically significant deterioration occurs.

Closed-loop sedation systems, which automatically adjust drug delivery based on physiological and electroencephalographic parameters, represent a promising approach to improving precision and reducing the risk of over- and under-sedation. However, further studies are needed to evaluate their safety, feasibility, and cost-effectiveness in endoscopic settings.

In parallel, the development of drug-specific monitoring algorithms that account for the distinct electrophysiological signatures of different sedative agents may enhance the accuracy of pEEG interpretation, particularly for non-GABAergic drugs such as ketamine and dexmedetomidine.

Future clinical trials should prioritize high-risk populations, including elderly patients, those with significant cardiopulmonary comorbidities, and patients undergoing prolonged or complex procedures, as these groups are most likely to benefit from advanced and individualized approaches.

In addition, cost-effectiveness and implementation studies are required to define the optimal balance between monitoring complexity and clinical benefit, ensuring that advanced technologies can be integrated into routine practice without unnecessary resource utilization. These developments will be essential to support the transition toward precision sedation and individualized monitoring strategies in gastrointestinal endoscopy.

## 11. Conclusions

Monitoring the depth of sedation during gastrointestinal endoscopy remains a critical component of patient safety and procedural success. Clinical assessment, including validated sedation scales and continuous observation, remains the foundation of monitoring, particularly for moderate sedation. However, these methods are intermittent and may fail to detect early physiological deterioration.

Objective monitoring technologies provide complementary information. Capnography allows early detection of respiratory compromise and may reduce the incidence of hypoxemia, particularly during deep sedation and in high-risk patients. Processed electroencephalogram (pEEG)-based systems, such as bispectral index and entropy, allow continuous assessment of sedation depth, although their interpretation is influenced by the drug-specific effects and their impact on clinical outcomes remains inconsistent.

Current evidence supports a tailored approach to sedation monitoring, considering the depth of sedation, type of procedure, and patient-related risk factors. No single modality is sufficient in isolation; rather, the integration of clinical assessment with cardiopulmonary and selected advanced monitoring techniques provides the most effective strategy.

Clinical assessment remains the cornerstone of sedation monitoring during gastrointestinal endoscopy, particularly for moderate sedation. Capnography should be considered during deep sedation in patients at increased risk of respiratory compromise, as it enables earlier detection of hypoventilation and apnea compared to pulse oximetry alone. Processed electroencephalogram (pEEG)-based monitoring may provide additional value in selected cases, particularly during propofol-based sedation and in high-risk or prolonged procedures, but should not replace clinical assessment. The proposed multimodal approach, illustrated in Figure 1 and Figure 2, provides a practical framework for selecting monitoring strategies according to sedation depth, procedural complexity, and patient-specific risk factors. This framework facilitates the integration of clinical assessment with pEEG-based monitoring and supports individualized decision-making in routine endoscopic practice.

## Figures and Tables

**Table 1:** Recommended monitoring strategies according to the depth of sedation during gastrointestinal endoscopy.

Sedation Level	Typical Agents	Monitoring Required	Additional Considerations
Minimal sedation	Low-dose benzodiazepines	Clinical observation, pulse oximetry	Very low risk
Moderate sedation	Midazolam ± opioids	Clinical scales (RASS/OAA-S), pulse oximetry, blood pressure	Standard practice
Deep sedation	Propofol	Capnography, ECG, continuous monitoring	Increased risk of respiratory depression
General anesthesia	Propofol ± adjuncts	Full monitoring (capnography, ECG, pEEG)	Requires anesthesia expertise

**Table 2:** Comparison of monitoring modalities for assessing sedation depth during gastrointestinal endoscopy.

Monitoring Modality	Parameters Assessed	Advantages	Limitations	Level of Evidence
Clinical scales (RASS, OAA/S)	Sedation depth (clinical)	Simple, no cost, widely used	Intermittent, subjective	Moderate
Pulse oximetry	Oxygen saturation	Non-invasive, standard of care	Late detection of hypoventilation	High
Blood pressure monitoring	Hemodynamic status	Essential safety parameter	Intermittent	High
Capnography	Ventilation (ETCO_2_)	Early detection of apnea/hypoventilation	Cost, false positives	Moderate–High
ECG monitoring	Cardiac rhythm	Detects arrhythmias	Limited role in low-risk patients	Moderate
BIS (bispectral index)	EEG-derived sedation depth	Continuous, objective	Drug-dependent variability	Moderate
Entropy/PSI	EEG-derived sedation depth	Alternative to BIS	Less validated in endoscopy	Limited–Moderate

## Data Availability

The original contributions presented in this study are included in the article. Further inquiries can be directed to the corresponding authors.
